# Beziehungsgeschichten. Kunstaffine Ärzt:innen, begabte Künstlerpatient:innen und die Debatte um Kunst aus psychiatrischem Kontext nach 1945

**DOI:** 10.1007/s00115-023-01592-3

**Published:** 2024-01-11

**Authors:** C. Beyer, C. F. Gümpel, T. Röske, M. Rotzoll

**Affiliations:** 1 Institut für Geschichte der Pharmazie und Medizin, Philipps-Universität Marburg, Roter Graben 10, 35037 Marburg, Deutschland; 2 Sammlung Prinzhorn, Klinik für Allgemeine Psychiatrie, Universitätsklinikum Heidelberg, Voßstraße 2, 69115 Heidelberg, Deutschland

**Keywords:** Kunst, Nachkriegszeit, Arzt-Patienten-Beziehung, Kreativität, Art brut, Art, Postwar Europe, Doctor-patient relationship, Creativity, Outsider art

## Abstract

**Hintergrund:**

1945 prägte der Künstler und Sammler J. Dubuffet den Begriff Art brut u. a. für originelle Werke von Psychiatrieinsassen, die außerhalb von Traditionen und Kunstströmungen entstanden waren. In den Jahrzehnten danach standen diese Werke im Zentrum von Aushandlungsprozessen, in die sich neben Psychiater:innen nun auch verstärkt Ausstellungsmacher:innen, Galerist:innen etc. einmischten.

**Fragestellung:**

Anhand von vier „Beziehungsgeschichten“ zwischen Psychiater:innen und Künstlerpatient:innen (H. Müller-Suur – P. Goesch; M. in der Beeck – E. Spießbach; J. Porret-Forel – A. Corbaz; L. Navratil – R. Limberger) werden schlaglichtartig Herangehensweisen an das Spannungsfeld Kunst und Psychiatrie nach 1945 dargestellt.

**Material und Methoden:**

Die dargestellten Ergebnisse des Teilprojektes „Normal#verrückte Kunst. Werke aus psychiatrischem Kontext zwischen Diagnostik und Ästhetik nach 1945“ der DFG-Forschungsgruppe „Normal#Verrückt“ (FOR 3031) werden durch die Auswertung von Archivbeständen, Nachlässen, Zeitzeug:inneninterviews und zeitgenössischen Medien erzielt.

**Ergebnisse:**

Es wird gezeigt, dass die unterschiedliche Haltung der genannten Psychiater:innen zu „ihren“ Künstlerpatient:innen deren Eingang in die Kunstwelt stark beeinflussten. Wichtig waren dabei v. a. Impulse jenseits der Psychiatrie, um rein diagnostische Sichtweisen auf die Werke durch andere Zugänge zu erweitern.

**Diskussion:**

Das erneute Interesse an der individuellen Kreativität von Patient:innen nach 1945 kann als Reaktion auf ihre Entmenschlichung im Faschismus und Nationalsozialismus verstanden werden. Jedoch konnte die Konzentration auf die pathologisierte Persönlichkeit der Künstlerpatient:innen alternative Blickwinkel auf ihre Kunst verstellen, wie auch die Verfügung über ihre Werke durch Psychiater:innen ihre Verbreitung behindern konnte.

## Abstract (en)

**Background:**

In 1945 the artist and art collector J. Dubuffet coined the term Art brut for original works by psychiatric inmates that had been created outside of traditions and art movements. In the following decades these works were at the center of negotiation processes in which not only psychiatrists but also exhibition organizers, gallery owners etc. increasingly became involved.

**Objective:**

Based on the evaluation of four exemplary pairs of psychiatrists and artist patients (H. Müller-Suur—P. Goesch; M. in der Beeck—E. Spießbach; J. Porret-Forel—A. Corbaz; L. Navratil—R. Limberger), this study explores the field of tension between art and psychiatry after 1945.

**Material and methods:**

The results of the subproject “Normal#Crazy Art. Works from a Psychiatric Context between Diagnostics and Aesthetics after 1945” of the German Research Foundation (DFG) research group “Normal#Crazy” (FOR 3031) are based on the evaluation of archival material, estates, interviews with contemporary witnesses and contemporary media.

**Results:**

It is shown that different attitudes of the psychiatrists towards “their” artist patients strongly influenced their entry into the art world. In this context, impulses from beyond psychiatry were important in order to expand purely diagnostic views of the works with other approaches.

**Discussion:**

The renewed interest in the individual creativity of patients after 1945 can be understood as a reaction to their dehumanization under fascism and National Socialism; however, the focus on the pathologized personality of artist patients could obscure alternative perspectives on their art, just as the disposal of their works by psychiatrists could hinder their dissemination.

## Hintergrund und Fragestellung

Nach dem Zweiten Weltkrieg ist eine kontinuierliche Erosion der psychiatrischen Deutungshoheit über das kreative Schaffen von Menschen mit psychischen Ausnahmeerfahrungen festzustellen. 1945 prägte der Künstler und Sammler Jean Dubuffet den Begriff „Art brut“ für originelle Werke von Laien, die außerhalb von Traditionen und aktuellen Kunstströmungen entstanden waren, die er jedoch für die eigentliche Kunst hielt, darunter stark vertreten Werke von Psychiatriepatient:innen. Damit bezog er dezidiert Stellung im Diskurs über die Zuordnung dieser Werke zwischen Pathologisierung und Aufwertung zur Kunst. Die Debatte hatte bereits vor dem Zweiten Weltkrieg mit Hans Prinzhorns „Bildnerei der Geisteskranken“ begonnen, bis sie im deutschen Sprachraum mit dem Nationalsozialismus fast vollständig verdrängt worden war. Nach 1945 standen jedoch die entsprechenden Werke im Zentrum immer wieder neuer Aushandlungsprozesse, an denen sich neben Psychiater:innen nun auch verstärkt Ausstellungsmacher:innen, Galerist:innen, Sammler:innen, Journalist:innen, Kunsttherapeut:innen und nicht zuletzt die Künstler:innen selbst beteiligten.

Der Übergang solcher Werke aus einer vor allem psychiatrischen Perspektive in die der Kunstwelt war, so unsere Hypothese, auch durch das Agieren kunstaffiner Ärzte, seltener Ärztinnen bestimmt, die über diese Werke verfügten: Ihr Einsatz oder dessen Fehlen konnte entscheidend dafür sein, ob die Werke „ihrer“ Patient:innen Eingang in die Kunstwelt fanden. Deren Rolle soll im Beitrag anhand der vier wichtigsten Akteure in diesem Feld nachgezeichnet werden, die sich in besonderer Weise jeweils einem/r Künstlerpatient:in widmeten: Hemmo Müller-Suur (1911–2001) und Paul Goesch (1885–1940), Manfred in der Beeck (1920–2004) und Erich Spießbach (1901–1956), Jacqueline Porret-Forel (1916–2014) und Aloïse Corbaz (1886–1964) sowie Leo Navratil (1921–2006) und Rudolf Limberger (1937–1988). Andere Psychiater der Zeit befassten sich zwar ebenfalls mit künstlerisch tätigen Anstaltsinsassen, bauten aber keine solch enge Beziehung zu ihrer Person und/oder ihrem Werk auf. Herausragendes Beispiel hierfür ist Helmut Rennert (1920–1994), Lehrstuhlinhaber in Halle seit 1958, der das Interesse an Kunst von seinem Vorgesetzten Rudolf Lemke (1906–1957) übernommen hatte [12], in seinen eigenen Publikationen aber nur distanziert diagnostisch vorging [26, 27].

Die folgenden vier chronologisch geordneten „Beziehungsgeschichten“ verdeutlichen schlaglichtartig unterschiedliche Herangehensweisen an das Spannungsfeld Kunst und Psychiatrie nach 1945. Dabei liegt der Kristallisationspunkt in den ersten Nachkriegsjahren.

## Studiendesign und Untersuchungsmethoden

Der Beitrag speist sich aus Ergebnissen des Forschungsprojektes „Normal#verrückte Kunst. Werke aus psychiatrischem Kontext zwischen Diagnostik und Ästhetik nach 1945“, das in der seit 2022 arbeitenden DFG-Forschungsgruppe „Normal#Verrückt. Zeitgeschichte einer erodierenden Differenz“ angesiedelt ist. Wesentliches Projektziel ist es, das Nachzeichnen der Erosionsprozesse zwischen Kunst, Kunstmarkt und Kunstbetrieb einerseits sowie Psychiatrie, Psychiatrieerfahrung und psychischem Ausnahmezustand andererseits für die Zeitgeschichte des Psychischen fruchtbar zu machen. Dies geschieht durch die erstmalige Auswertung umfangreicher Archivbestände und Nachlässe (u. a. die nachgelassene Korrespondenz von Leo Navratil in der Österreichischen Nationalbibliothek Wien, die Sammlungen Manfred in der Beeck und Hemmo Müller-Suur im Archiv der Sammlung Prinzhorn Heidelberg sowie der Nachlass des Galeristen Daniel Cordier in der Bibliothèque Kandinsky im Centre Pompidou Paris), durch Zeitzeug:inneninterviews mit psychiatrisch Tätigen, Galerist:innen und Ausstellungsmacher:innen sowie durch die Analyse der Rezeption von Künstlerpatient:innen in Presse, Fachzeitschriften und Rundfunk.

## Kunst als Illustration der Psychopathologie und späte Wertschätzung – Hemmo Müller-Suur und Paul Goesch

An der Göttinger Universitätsklinik befasste sich Ende der 1940er-Jahre der Psychiater Hemmo Müller-Suur mit der Kunst des ehemaligen Patienten und Architekten Paul Goesch. Der in avantgardistischen Künstlerkreisen verkehrende Goesch erlebte seine erste nervliche Krise 1909 und wurde schließlich 1921 in die Göttinger Psychiatrie mit der Diagnose „Dementia praecox“ bzw. „Schizophrenie“ aufgenommen [28]. Den Künstler verlegte man 1934 in die Anstalt Teupitz, Müller-Suur kam 1938 als Assistenzarzt nach Göttingen. Der Psychiater lernte deshalb nur die Werke Goeschs und nicht den Künstlerpatienten selbst kennen, der 1940 ermordet wurde [30]. Mehr als 300 seiner spielerischen, zwischen Expressionismus und Abstraktion stehenden Werke befanden sich über fünfzig Jahre im Besitz Müller-Suurs, bevor sie schließlich nach dessen Tod einem Großneffen des Künstlers übergeben wurden [16]. Müller-Suur machte durch Präsentationen seiner Sammlung, z. B. auf der Wanderausstellung *Documenta Psychopathologica* 1966, Goeschs Werke für ein breiteres Publikum zugänglich.

Es ist wahrscheinlich, dass Müller-Suur als Assistenzarzt von Gottfried Ewald (1888–1963; [5]) erstmals mit Zeichnungen Goeschs in Berührung kam. Ewald veröffentlichte in seinem *Lehrbuch der Neurologie und Psychiatrie* 1944 zwei Aquarelle Goeschs, die er als Produkte eines unkontrollierbaren Schreib- oder Mal-Drangs im Zustand der Schizophrenie interpretierte. Dieser pathologisierende Blick hinderte den Arzt jedoch nicht daran, den Werken einen „künstlerischen Wert“ zuzusprechen [8, S. 373].

**Figure 1:**
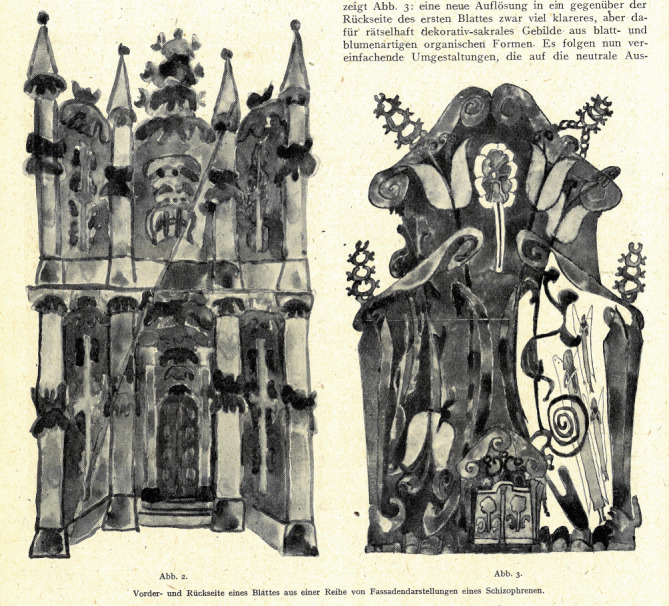

Höchstwahrscheinlich übernahm Müller-Suur nach Ewalds Emeritierung 1954 das große Konvolut an Werken. Er beschäftigte sich mit Goesch bereits 1948, als er in einem Artikel vier Aquarelle als Paradebeispiele für „schizophrene Kunst“ analysierte. Der Psychiater erkannte den ästhetischen Reiz der Bilder, schrieb jedoch den Beginn ihrer Produktion ausschließlich einer akuten Episode visueller und akustischer Halluzinationen zu: Der Eintritt in den psychotischen „mundus fabulosus“ sei hauptsächlich dem Psychiater vorbehalten, da dieser am besten „die Grenze der Ausprägung schizophrener Kriterien“ feststellen könne. Obwohl die abgebildeten Aquarelle vom Künstler selbst nicht datiert worden waren, sah er in der „Evolution“ der Motive den Zerfall des Künstlers im „schizophrenen Krankheitsprozess“ ([13, S. 154–157]; Abb. 1).

Müller-Suur analysierte die Zeichnungen Goeschs auch aus existenziell-anthropologischer Perspektive und verglich sie 1967 mit Werken der klassischen Moderne [14]. Seine Näherung „schizophrener Kunst“ an „Hochkunst“ zeigt auch ein Katalogbeitrag von 1976 zu Adolf Wölfli, in dem das „Künstlersein“ des Patienten Wölfli nicht mehr seiner Erkrankung untergeordnet wird [15, S. 105]. Doch besonders die Stiftung von acht Werken Paul Goeschs an die Hamburger Kunsthalle 1967 macht ein Umdenken des Psychiaters deutlich. So kann man sich fragen, ob Müller-Suurs Abwendung von einer rein psychopathologischen Betrachtung nicht auch mit der Neubewertung von Werken Psychiatrieerfahrener in den 1960er- und 1970er-Jahren zusammenhängt, die durch Ausstellungen wie die *documenta 5* und durch die Schriften von Dubuffet und Roger Cardinal [7] eine breitere Akzeptanz dieser Werke als Kunst *tout court* bewirkte.

## Ein „AHA-Erlebnis“ – Manfred in der Beeck und Erich Spießbach

Die Begegnung mit Erich Spießbach in den Jahren 1951/1952 bezeichnete Manfred in der Beeck retrospektiv als „AHA-Erlebnis künstlerischer Therapie“, das am Anfang seines „Wegs in die Psychopathologie des Ausdrucks“ gestanden habe [4, S. 10]. Auf diesem Weg profilierte sich der Psychiater früh als Gründungsmitglied der Société Internationale de Psychopathologie de l’Expression (SIPE) 1959 und der Deutschsprachigen Gesellschaft für die Psychopathologie des Ausdrucks (DGPA) 1964.

Spießbach arbeitete ab 1928 am Museum für Ur- und Frühgeschichte in Münster. Anhaltende Streitigkeiten mit seinen Vorgesetzten mündeten 1936 in seine Entlassung. Beleidigungen und Anschuldigungen auch gegenüber Behörden führten zu seiner Entmündigung und 1943 zu einer Einweisung als „querulierender Paranoiker“ in die Anstalt Münster, von wo er bald nach Marsberg gebracht wurde [29]. Wegen einer Tuberkuloseerkrankung isoliert, traf Spießbach dort auf den damaligen Assistenzarzt in der Beeck. Dieser versorgte ihn zur Beschäftigung mit Zeichenmaterial. Die Vorlage der Sentenz: „Alles ist möglich, das Dümmste aber am wahrscheinlichsten“, führte zu einer intensiven, wenige Wochen dauernden kreativen Phase Spießbachs, in der er über 300 figurative Zeichnungen anfertigte, die sich ironisch bis sarkastisch der Dummheit anderer, nicht zuletzt der Psychiater, widmen. Als er jedoch anfing, Bettlaken mit kommunistischer Symbolik an die Türen und Fenster seines Einzelzimmers zu drapieren, verlegte ihn in der Beeck zurück auf eine Wachstation. Daraufhin weigerte sich Spießbach weiter zu zeichnen. Im April 1953 wurde er wieder in die Anstalt Münster gebracht. Seine Werke blieben laut Patientenakte in Marsberg zur „wissenschaftlichen Bearbeitung“. Spießbach verunfallte im Oktober 1956 bei einem Ausbruchsversuch tödlich.

**Figure 2:**
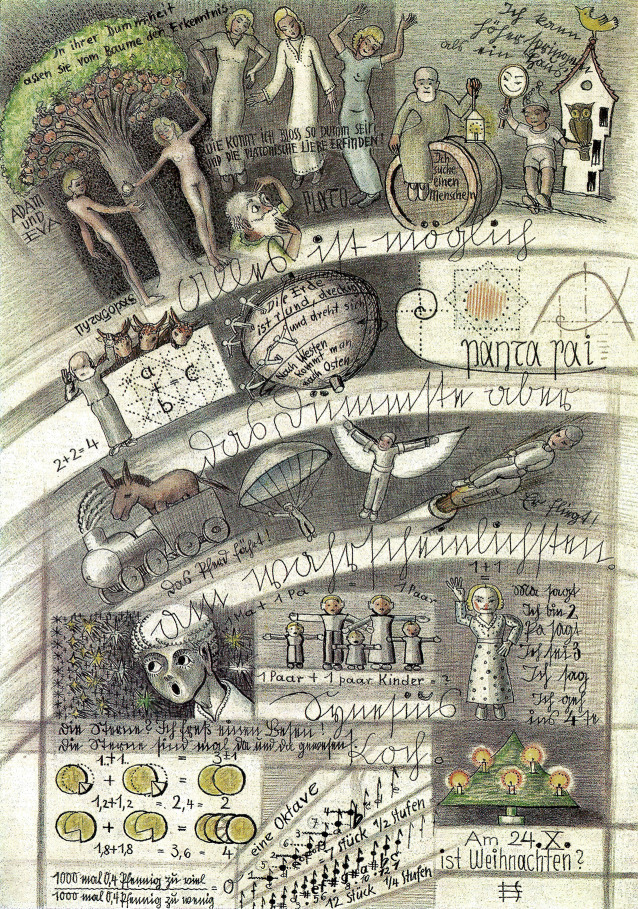

Spießbachs Werke betrachtete in der Beeck offensichtlich als seinen Privatbesitz, und so nahm er sie 1959 in das Landeskrankenhaus Schleswig mit, wo er bis zu seiner Pensionierung tätig war. Die angekündigte „wissenschaftliche Bearbeitung“ der Werke erfolgte allerdings kaum. 1961 erschien die Zeichnung „Alles ist möglich“ in einer Zeitschrift der Pharmafirma Boehringer. In der Beeck charakterisierte sie dort lediglich knapp als Beispiel „veränderter Vorstellungen und Denksysteme der erkrankten Persönlichkeit“ ([2]; Abb. 2).

Im Jahr 1966 brachte das Pharmaunternehmen Bayer einen Band mit Werken Spießbachs in einer Auflage von 15.000 Exemplaren heraus. Darin verzichtete in der Beeck bewusst auf eine Interpretation der Werke: Er wollte die „psychopathologisch aufschlussreiche[n] Schöpfungen“ den Leser:innen „wertfrei“ vorstellen, schrieb ihnen aber eine „begnadete humorige Note“ zu und fragte, ob es „schizophrenen Humor“ gebe [1]. Dieses Konzept blieb aber ebenso unausgearbeitet wie in der Beeck andere Perspektiven auf das Werk Spießbachs unberücksichtigt ließ, z. B. die Thematisierung der Beziehung des Zeichners zu seinem Psychiater.

Die Werke Spießbachs waren deshalb bis zu ihrer Übernahme in die Sammlung Prinzhorn 2017 einem kleinen Kreis kunstinteressierter Psychiater:innen vorbehalten – den Weg in die Kunstwelt schafften sie nicht. Präsentationen bei internationalen Fachtagungen und Vorträgen etwa in Paris und Washington in den 1960er-Jahren erzielten keine nennenswerte Resonanz außerhalb dieser Fachkreise [4, S. 95, 159, 165–166]. Bis zum Ruhestand in der Beecks lassen sich keine Anhaltspunkte für Anstrengungen von ihm finden, Spießbach in der Kunstwelt zu verankern. Das wachsende Interesse an „Outsider Art“ kritisierte er als Modeerscheinung: „[…] Außenseitertum [wurde] modern, ‚out‘ wurde ‚in‘, und man konnte sich vor Anforderungen, ‚Irrenkunst‘ zu vermarkten, nicht retten“ [3].

## Zu zweit in einer magischen Welt – Jacqueline Porret-Forel und Aloïse Corbaz

Jacqueline Porret-Forel aus Morges (CH), entfernte Verwandte des Psychiaters Auguste Forel (1848–1931), studierte Medizin und wurde Hausärztin [25]. Für ihr Arbeitsleben wurde die Begegnung mit der Patientin Aloïse Corbaz entscheidend: Hans Steck (1891–1980), Leiter der Anstalt La Rosière/Gimel, hatte deren Zeichnungen in klinischen Kursen vorgestellt [21, S. 6, 9]. Porret-Forel entwickelte eine enge Beziehung zu Corbaz, die sich von den distanzierten Beziehungen ihrer männlichen Kollegen zu „ihren“ Künstlerpatienten deutlich unterschied. Porret-Forel widmete Corbaz nicht nur ihre Dissertation von 1953, sondern auch alle weiteren Publikationen. Dabei stellte sie die „Schizophrenie“ als bedeutsamen Faktor für die Werke der Patientin nie infrage, arbeitete jedoch bald mit Dubuffet zusammen, den sie 1947 mit dem Werk von Corbaz bekannt machte. Mit einer gemeinsamen Publikation zu Corbaz avancierte diese zur „Art-brut“-Künstlerin *par excellence*. Ihr Werk ist bis heute zentraler Bestandteil der *Collection de l’Art Brut* in Lausanne.

**Figure 3:**
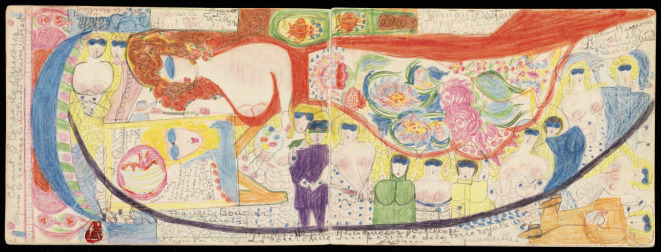

Corbaz wurde in Lausanne geboren. Nach einer Schneiderinnenlehre wurde sie 1911 angeblich Gouvernante im Haushalt des Kaplans von Kaiser Wilhelm II., zu dem sie Gefühle von Liebe und Verehrung entwickelte [6, S. 71]. In die Schweiz zurückgekehrt, wurde sie 1918 in die Anstalt Céry/Lausanne eingewiesen. Von 1920 bis zu ihrem Tod 1964 lebte Corbaz in der ländlichen Anstalt La Rosière/Kanton Waadt [10]. Soweit bekannt, begann sie erst dort zu malen und zu zeichnen. Heute ist sie weithin bekannt für ihre farbstarken großformatigen Kreidezeichnungen, die vor allem erotisch aufgeladene Paarbeziehungen zeigen, aber in ihren vielfältigen symbolischen Bezügen schwer zu entschlüsseln sind – Stoff genug für Porret-Forels Lebenswerk (Abb. 3).

Ärztin und Patientin begegneten sich erstmals 1941 in der Anstalt La Rosière. Für die Jahre danach beschreibt Porret-Forel eine Verhaltensänderung bei Corbaz, die sie auf ihre Kreativität zurückführt. So sei diese nicht mehr so „explosiv“ und könne kohärent über ihre Zeichnungen Auskunft geben. Einerseits habe die künstlerische Arbeit eine neue Verbindung zur materiellen Welt hergestellt, andererseits konkretisierten die Werke das wahnhafte Erleben und ermöglichten es so, andere daran teilhaben zu lassen. Insofern betont die Ärztin therapeutische Aspekte und insbesondere den kommunikativen Aspekt, ohne sich selbst als langjährige Gesprächspartnerin Corbaz’ zu thematisieren.

Zu Werkinhalten finden sich in der Dissertation Porret-Forels bereits die einordnenden Begriffe, die sie später beibehält: Es geht um ein „Universum“ mit symbolisch aufgeladenen Personen, um theatrale Inszenierung, um das Erschaffen einer magischen Welt zur Krankheitsabwehr [21, S. 17–18, 27, 41, 22, S. 28]. Ihre Schlussfolgerung: „Wir haben versucht, darzustellen, wie eine paranoide Schizophrene die von ihrem Wahn erzeugte Welt in Zeichnungen umsetzte und ihr Leben einhauchte. Wir wollten die Fortschritte aufzeigen, die sie machte, als sie die starre Form, die ihr durch die Psychose auferlegt worden war, durch die Kunst sprengte“ [21, S. 62].

Porret-Forel ließ sich auf dieses Universum ein und lebte auch nach dem Tod der Künstlerin umgeben von deren Bildern. „Ihr Werk hat meine geistige Landschaft geprägt“, fasste sie 1993 zusammen [23, S. 22]. 2004 gab sie die schriftlichen Zeugnisse von Corbaz heraus, 2012 folgte ein Œuvre-Katalog. Zugleich trug Porret-Forel, nun 96 Jahre alt, mehrere Katalogtexte zur Einzelausstellung „Aloïse. Le ricochet solaire“ in der *Collection de l’Art brut* bei [11]. Offenbar wurde sie nicht müde, die „Welt von Aloïse“ zu beschreiben und zu vermitteln [24].

## Vom Schizophrenietest auf den Kunstmarkt – Leo Navratil und Rudolf Limberger

Leo Navratil, seit 1946 Psychiater auf einer Männerstation der niederösterreichischen Heil- und Pflegeanstalt Gugging/Klosterneuburg, lernte im 1952/1953 bei einem Studienaufenthalt in London das Buch *Personality Projection in the Drawing of the Human Figure* (1949) der amerikanischen Psychologin Karen Machover kennen [19, S. 117]. Zurück in Gugging 1954 bat er, davon angeregt, jeden neu aufgenommenen Patienten, in seiner Anwesenheit eine menschliche Figur mit Bleistift auf einen postkartengroßen weißen Karton zu zeichnen. Bald ließ er die Patienten diese Prozedur täglich wiederholen, verglich die Ergebnisse und zog diagnostische Schlüsse [18, S. 5]. Er las die klassischen Veröffentlichungen von Walter Morgenthaler (1921) und Hans Prinzhorn (1922) über künstlerische Schöpfungen von Psychiatrieinsassen, und der Schweizer Psychiater Alfred Bader machte ihn ab 1958 mit weiteren zeichnenden Patient:innen der Psychiatrien von Céry und Lausanne bekannt (u. a. Aloïse Corbaz).

Wichtige Anregung für ihn waren zudem die Bücher *Die Welt als Labyrinth. Manierismus in der europäischen Kunst und Literatur* (1957) und *Manierismus in der Literatur* (1959), in denen der Literaturwissenschaftler Gustav R. Hocke seine Idee eines zyklisch wiederkehrenden Manierismus erläutert. Sie gaben Navratil eine Grundidee für sein Buch *Schizophrenie und Kunst* (1965) ein, in dem er die Zeichnungen ungeübter Psychiatriepatienten zur „Urgebärde des Manierismus“ erklärt. So konnte er trotz seiner Auffassung, dass die Werke „der Kranken aus der Psychose entspringen“ und hier die Psychose „der Künstler“ sei [17, S. 108, 6, 39], einen Vergleich zu professioneller Kunst herstellen.

Das Taschenbuch wurde schnell populär. Der Psychiater lernte Wiener Künstler kennen, die ihn dazu brachten, ab 1970 Werke seiner Schützlinge in Galerien und Ausstellungshäusern zu zeigen. Die Gruppe der zeichnenden Patienten wurde bald weit über die Grenzen Österreichs bekannt, 1980 übergab Navratil seine Sammlung ihrer Werke an die Neue Galerie der Stadt Linz, und 1981 richtete er für sie ein eigenes „Zentrum für Kunst-Psychotherapie“ auf dem Gelände der Gugginger Psychiatrie ein, das sein Nachfolger ab 1986, Johann Feilacher, zum „Haus der Künstler“ umbenannte.

**Figure 4:**
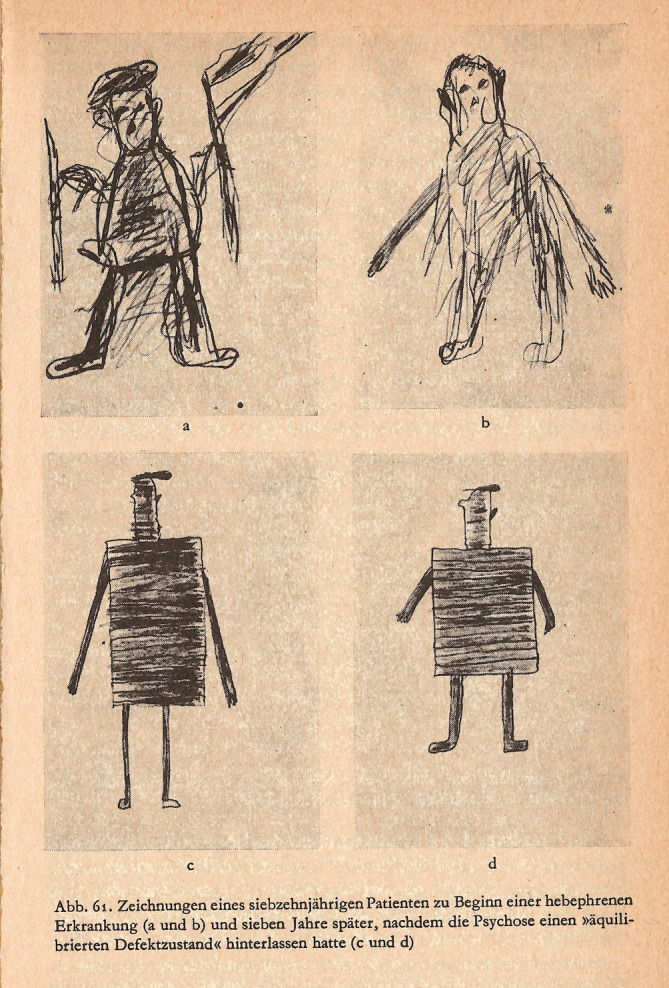

Zu den kreativen Patienten Navratils gehörte Rudolf Limberger, der bereits mit 16 Jahren nach Gugging kam [20, S. 447–448] und 1955 unter den ersten war, die für den Psychiater zeichneten. Bei seiner Wiederaufnahme mit 23 Jahren setzte er dies fort. Navratil stellt in *Schizophrenie und Kunst* (1965) zwei frühe Zeichnungen Limbergers zwei späteren gegenüber. Erstere interpretiert er als „angsterfülltes deformiertes Menschenbild“ am „Beginn einer hebephrenen Erkrankung“, bei letzteren deutet er die „stereotype“ „Geometrisierung“ der dargestellten Körper als „Ergebnis eines Restitutionsversuches“ im „equilibrierten Defektzustand“ einer Psychose ([17, S. 74–76]; Abb. 4). Auch in der Folge zeichnete Limberger, der seit 1975 dauerhaft in Gugging lebte, häufig, allerdings immer nur im Beisein des Psychiaters und ohne Interesse am Ergebnis. Navratil sah zudem in seinen mit Hiebkritzeln gefüllten Menschenfiguren und anderen Darstellungen „mehr eine motorische Abreaktion“ und hob ihn von den anderen „Künstlern“ unter seinen Patienten ab. Dass er dies wertend meinte, kommt darin zum Ausdruck, dass er an seinem Pseudonym „Max“ festhielt, während er die meisten anderen seiner Schützlinge bereits mit ihrem Klarnamen benannte. Obwohl er bei Limbergers Zeichnungen Ähnlichkeiten zu den Übermalungen Arnulf Rainers sah, war es für ihn „fraglich, ob seine ‚Kritzeleien‘ Kunst wären“ [9]. Heute wird Rudolf Limberger unter seinem Klarnamen als einer der wichtigen Art-brut-Künstler Guggings gesehen, man schätzt sein vehementes, druckstarkes Füllen einfacher Formen als originelle Gestaltungen und seine Bilder erzielen steigende Preise auf dem Kunstmarkt.

## Diskussion

Müller-Suur, geprägt vom Blick seines Lehrers Ewald und wahrscheinlich auch aufgrund seiner anthropologischen und phänomenologischen Herangehensweise, erkannte die Produktionen Goeschs als künstlerisch wertvoll. Dennoch blieb seine ästhetische Einordung dieser Werke in einem stark pathologisierenden Rahmen. Die Ausstellungen Müller-Suurs, seine zahlreichen Publikationen und besonders die Schenkung 1967 führten allerdings zugleich zur erneuten Sichtbarkeit und schließlich zur Wiederentdeckung von Goeschs Werk. Dies dürfte auch mit einer Perspektivenveränderung auf diese Kunst in den 1960er-Jahren zusammenhängen, die Müller-Suur selbst durch die ausführliche Auseinandersetzung mit dem Normalitäts‑, Schizophrenie- und Kunstbegriff begleitete.

In der Beeck regte Spießbach zur Produktion seiner Werke an, für die er sich ebenfalls vorwiegend im diagnostischen Kontext interessierte. Er betrachtete sie wie damals üblich als seinen Privatbesitz, und seine rege Ausstellungstätigkeit nach der Pensionierung in Schleswig weist darauf hin, dass er selbst die Verfügungsgewalt über seine Sammlung und damit über das Werk seines längst verstorbenen Patienten Spießbach behalten wollte. Die Präsentationen von dessen Arbeiten erreichte zuvor – im Unterschied zu den anderen Beispielen – nicht die Kunstwelt. Im Gegensatz zu den anderen vorgestellten Ärzt:innen blieb eine eingehende inhaltliche Beschäftigung mit dem Werk „seines“ Patienten aus.

Früher als die Werke des Patienten Goesch und erst recht Spießbachs gelangten Werke der Schweizer Künstlerpatientin Aloïse Corbaz in Kunstausstellungsräume – so waren Arbeiten von ihr bereits Teil einer Ausstellung in der Kunsthalle Bern 1963. Dies war einerseits Jean Dubuffet zu verdanken, andererseits der Ärztin Jacqueline Porret-Forel. Sie interessierte sich offenbar nicht für diagnostische Kriterien im Umgang mit Kunst aus psychiatrischem Kontext, sondern eher für therapeutische Effekte aus dem Akt der Gestaltung. Dabei zweifelte sie bei Corbaz weder am Vorliegen einer „Schizophrenie“ noch am Kunstcharakter der Werke. Die Bedeutung der – von Seiten der Ärztin – fast symbiotisch anmutenden Beziehung zu der Künstlerin für deren Kunst wird wie bei in der Beeck nicht explizit thematisiert. Im Unterschied zu dem Psychiater ließ sich Porret-Forel dauerhaft auf die „Welt“ der Künstlerin ein und lieferte über Jahrzehnte tiefgreifende Analysen. Einige Jahre vor Navratil begann sie damit, eine einzige Künstlerpatientin letztlich weltbekannt zu machen. Auch wenn oder vielleicht gerade weil die psychische Krankheit für sie nicht infrage stand, trug sie damit wesentlich zur Erosion der Kategorien gesund und krank in der Kunst bei.

Der österreichische Psychiater Leo Navratil setzte sich nach Erscheinen seines Buches *Schizophrenie und Kunst* (1965) erfolgreich für die Ausstellung und Vermarktung von Patienten aus der Heil- und Pflegeanstalt Gugging ein, deren Kreativität er seit Mitte der 1950er-Jahre beim Anwenden eines Figurzeichentests entdeckt hatte. Die von ihm erzeugte Reichweite in den populären zeitgenössischen Kunstdiskurs unterscheidet ihn von seinen Kolleg:innen, insbesondere von in der Beeck. Obgleich er Anfang der 1980er-Jahre für diese Gruppe sogar ein eigenes „Zentrum für Kunst-Psychotherapie“ eingerichtet hat und seine eigene Sammlung ihrer Werke der Neuen Galerie Linz schenkte, haderte er weiterhin mit der Einordnung ihrer Werke als Kunst. Das zeigt sich insbesondere an seiner Haltung zu Rudolf Limberger, in dessen Werken er vor allem Symptome sah und dem er anhaltend das Pseudonym „Max“ gab.

## Schlussfolgerungen

Es wurde gezeigt, auf welche Weise sich vier Ärzt:innen Künstler:innen mit Psychiatrieerfahrung widmeten, in welcher Weise sie diese pathologisierten, aber auch, welchen Anteil sie an deren Eingang in die Kunstwelt und somit an der Erosion der Kategorien „normal“ und „verrückt“ hatten. Hierfür spielten Impulse von außerhalb des psychiatrischen Feldes eine wichtige Rolle, rein diagnostische Perspektiven auf das Werk von Patient:innen für Perspektiven der Kunstwelt zu öffnen. Die zeitliche Nähe dieses neu erwachten Interesses an Kunst aus psychiatrischem Kontext zu den Verbrechen des Nationalsozialismus legt nahe, dass die Hinwendung zur Kreativität von Patient:innen auch vor dem Hintergrund dieses Zivilisationsbruches zu verstehen ist: Das Verständnis von „Art brut“ als „eigentlicher Kunst“ (Dubuffet) und die psychiatrische Hinwendung zum individuellen Ausdruck von Patient:innen hingen historisch gemeinsam an der Frage, was „normale“ Kunst und Psychiatrie nach 1945 noch sein konnten.

## References

[1] Bayer Leverkusen – Pharmazeutisch-wissenschaftliche Abteilung, in der Beeck M (Hrsg) (1966) Wahnsinn, Ironie und tiefere Bedeutung. Bayer, Leverkusen

[2] in der Beeck M (1961). Psychisch Kranke, wie ein Maler sie sieht. Therapie des Monats 3.

[3] in der Beeck M (1980) Wahn – Sinn und Kunst. Zur Wanderausstellung der Prinzhorn-Sammlung: „Rebulutionsglaube verschwinde“, DÄ 30:1872–1884

[4] in der Beeck M (1988). Marginalien zur Psychiatrie.

[5] Beyer C (2013). Gottfried Ewald und die „Aktion T4“ in Göttingen. Nervenarzt.

[6] Brand-Claussen B, von Beyme I, Röske T (2022). Kaiserprojekte. Zu Leben und Werk von Else Blankenhorn und Aloïse. Else Blankenhorn – Das Gedankenleben ist doch wirklich. Ausst.-Kat., Sammlung Prinzorn, Das Wunderhorn.

[7] Cardinal R (1972). Outsider Art.

[8] Ewald G (1944). Lehrbuch für Neurologie und Psychiatrie.

[9] Feilacher J in einer E‑Mail an T Röske vom 29. Aug. 2022

[10] https://hls-dhs-dss.ch/de/articles/021972/2002-06-14/. Zugegriffen: 13. Sept. 2023

[11] Lepdor C, Marini-Jeanneret P (Hrsg) (2012) Aloïse, le ricochet solaire, Ausst.-Kat. Musée Cantonal des Beaux-Arts de Lausanne, Collection de l’Art brut Lausanne Bd. 5. Continents Edition, Lausanne/Mailand

[12] Lemke R (1958). Psychiatrische Themen in Malerei und Graphik.

[13] Müller-Suur H (1948). Schizophrene Kunst. Grenzgeb Med.

[14] Müller-Suur H (1967). Sinnhorizonte in Zeichnungen von Schizophrenen. Confinia Psychiatrica.

[15] Müller-Suur H, Adolf-Wölfli-Stiftung, Bern K (1976). Die Kunst Wölflis als Problem für die Psychiatrie. Adolf Wölfli. Ausst.-Kat.

[16] Müller-Suur N im Gespräch mit C F Gümpel, 18. Nov. 2022

[17] Navratil L (1965). Schizophrenie und Kunst. Ein Beitrag zur Psychologie des Gestaltens.

[18] Navratil L (1980). Die Geschichte der Sammlung und ihre Geschichtlichkeit. In: Neue Galerie der Stadt.

[19] Navratil L (1997). Leo.

[20] Navratil L (1997). Gugging 1946–1986. Bd. 2: Die Künstler und ihre Werke.

[21] Porret-Forel J (1953). Aloyse ou la peinture magique d’une schizoprène.

[22] Porret-Forel J (1978). Aloise. In: Third Eye Centre.

[23] Porret-Forel J (1993). Aloïse et le théâtre de l’univers.

[24] Porret-Forel J, Lepdor C, Marini-Jeanneret P (2012). Le monde d’Aloïse. Aloïse, le ricochet solaire, Ausst.-Kat. Musée Cantonal des Beaux-Arts de Lausanne, Collection de l’Art brut Lausanne.

[25] https://rawvision.com/blogs/obituaries/news-jacqueline-porret-forel. Zugegriffen: 13. Sept. 2023

[26] Rennert H (1966). Die Merkmale schizophrener.

[27] Rennert H (1977). Störungen der tiefenräumlichen Wahrnehmung und Wiedergabe. Ein europsychiatrischer Beitrag zur perspektivischen Erfassung, Leopoldina, Halle (Saale).

[28] Röske T (Hrsg) (2016) Paul Goesch 1885–1940. Zwischen Avantgarde und Anstaltskunst. Ausst.-Kat., Sammlung Prinzhorn. Das Wunderhorn, Heidelberg, S 10–14

[29] Röske T (2021). Ein Ausbruch in Kreativität. Erich Spießbach, „der dreifach diplomierte Idiot“. Das Wunderhorn.

[30] Rotzoll M, Röske T, Feise Mahnkopp P (2019). Endstation Brandenburg. Paul Goesch und der nationalsozialistische Krankenmord. „Von zweifellos künstlerischen Wert“. Paul Goeschs Beitrag zur ästhetischen Moderne.

